# Alexithymia among elderly patients with diabetes 

**Published:** 2013

**Authors:** Sevilay Hintistan, Dilek Cilingir, Nermin Birinci

**Affiliations:** 1 Sevilay Hintistan, RN, MS, PhD, Assistant Professor, Karadeniz Technical University Health Sciences Faculty, Nursing Department, Trabzon, Turkey.; 2 Dilek Cilingir, RN, MS, PhD, Assistant Professor, Karadeniz Technical University Health Sciences Faculty, Nursing Department, Trabzon, Turkey.; 3 Nermin Birinci, RN, Nurse, Vakfikebir State Hospital, Internal Clinic, Trabzon, Turkey.

**Keywords:** Alexithymia, Elderly, Diabetes Mellitus

## Abstract

***Objective:*** To determine alexithymic characteristics and affecting factors in the elderly with diabetes mellitus.

***Methods:*** This study was planned as a cross-sectional and descriptive study and was conducted in a state Hospital with 120 elderly patients with diabetes. Data were collected using a personal information form and the Toronto Alexithymia Scale.

***Results:*** Alexithymia was found in 75.8% of the elderly with diabetes mellitus. It was determined that patients experienced higher than average difficulty in describing and identifying feelings. They also had more externally-oriented thinking styles. Patients’ income levels, family structure and illness duration affected their manifestation of alexithymic characteristics.

***Conclusion: ***Majority of elderly patients with diabetes exhibited alexithymic characteristics. However, these were not shown to be associated with sex, age, marital, educational or professional status. In addition, no connection was found between alexithymia and glycosylated hemoglobin (HbA1c), body mass index (BMI), postprandial glucose (PBG), and the treatment and complications of diabetes mellitus.

## INTRODUCTION

Advancing age often brings physical, psychological and social changes as well as an increase in the occurrence of chronic diseases.^1^ One chronic and physical disease of the elderly population is diabetes mellitus Type II (DM), which often includes many psychiatric and psycho-social dimensions.^2^^,^^3^

The exact meaning of alexithymia is “without words for emotion.” This condition manifests itself as an inability to describe and identify emotions. Those afflicted exhibit an externally-oriented thinking style and a tendency to prefer the worlds of fantasy and imagination. Patients also experience difficulties with emotional functions and inter-personal relations in expressing and distinguishing feelings.^4^ Neuropsychological evidence demonstrates that some elderly may have problems processing emotions, and they are also less able to describe emotions such as anger and sorrow.^5^ Alexithymia is said to be associated with chronic diseases due to advanced age. Its characteristics are seen in about 4.7% of young people, whereas 29.3% of the elderly are affected.^6^^,^^7^ Sapozhnikova et al. (2012) examined 265 patients with Type 2 DM and 65 gender- and age-matched control individuals. When compared with the control group, patients with Type II DM had higher alexithymia levels (47.2% versus 21.5%).^8^

It has been reported that alexithymia is often associated with DM.^9^ Diabetes negatively affects not only the physical and social lives of the elderly but also their psychological well-being.^10^ A diagnosis of diabetes hinders the abilities of the elderly to live independently.^11^ Patients with diabetes experience many emotional reactions concerning their illness. When patients can verbally describe and identify their emotions, their psychological and physical symptoms may also improve.^7^ When emotions are not or cannot be verbally expressed, the body responds accordingly. The patient’s symptoms become somatic complaints, and the body will tend to show an increase in the alexithymic characteristics.^12^

It is estimated that one-third of patients who visit the diabetes centers are not properly diagnosed and treated. Although they present with symptoms, such as fatigue, sleep disorder, changes in appetite and weight, and loss of libido, these are seen in both psychiatric illnesses as well as diabetes. Consequently, a misdiagnosis means that the psycho-social dimension of the diabetes is overlooked.^2^ Therefore, emphasis should be laid on how alexithymic characteristics affect the treatment and care of the elderly DM patients occur. When these patients are able to accurately describe and identify their emotions, health care personnel will be better prepared to give the proper care and treatment needed.

The aim of the present study was to determine the alexithymic characteristics of the elderly with diabetes and the affecting factors of the alexithymic characteristics.

## METHODS

***Sample and*** ***Setting: ***This was a cross-sectional and descriptive study conducted at the endocrine policlinic of the state Hospital with 120 patients who had received outpatient treatment between March 7, 2011 and September 7, 2011. These patients had been diagnosed with Type II DM, were aged ≥ 60, did not have any psychiatric disease, had agreed to participate in the research and were able to answer the questionnaire questions.

***Data Collection and Instruments: ***A Personal Information Form (PIF) and the Toronto Alexithymia Scale (TAS-20) were used for the data collection.

***The PIF:*** This form was designed by researchers after a thorough review of the literature. The questionnaire had nine socio-demographic questions and six questions related to disease characteristics.

***Toronto Alexithymia Scale (TAS-20):*** The TAS-20 was developed by Bagby et al,^13^ and its Turkish validity and reliability tests were conducted by Sayar et al.^14^ As a self-rating scale, TAS-20 is a five point Likert-type scale and consists of 20 items. The TAS-20 has 3 subscales: the first subscale is Difficulty Identifying Feelings (DIF) “I have difficulty identifying the correct words for my emotions”; the second subscale is Difficulty Describing Feelings (DDF) “I have difficulty describing my emotions”; and the third subscale is Externally-Oriented Thinking (EOT) “I would rather talk about people’s daily life activities than their emotions”. Total score ranges from 20 to 100. Higher scores indicate a higher alexithymic level. There is a cut-off point used in the scale whereby scores ≥ 61 are accepted as alexithymic and scores ≤ 51 are accepted as non-alexithymic.^13^

***Procedure: ***Patients who agreed to participate in the study and met the inclusion criteria were asked to sign a written informed consent form. Data were collected by researchers through individualized interviews with patients at the endocrine policlinic. The patients were given the option of completing the questionnaire with or without assistance, and the illiterate patients chose to have the researchers read the questionnaires to them and record their responses. The patients were also given the chance to ask any question related to the study.

***Data Analysis: ***The data were analysed using the Statistical Package for Social Sciences (SPSS) version 11.0 for Windows (SPSS, Chicago, IL, USA). Descriptive data on frequency and percentage were used for the socio-demographic and DM-related variables. The Chi-square test was used to determine the association between socio-demographic and patients’ DM-related variables and their alexithymia characteristics. Mean and standard deviations were used for the TAS-20. The results were analyzed with a 95% confidence interval, and the accepted level of significance for all analyses was p*<*0*.*05.

***Ethical considerations:*** The ethical approval for the study was obtained from the hospital administration. The aim and method of the proposed study were carefully explained to the patients, and they were also informed that all information would be kept strictly confidential.

## RESULTS

A total of 120 patients were included in this study. Female participants comprised 65.8% of the group, and 45.8% belonged to the 70-79 age group. Seventy percent of patients were married; 35% were illiterate; 52.5% of the patients were housewives; 64.2% had a moderate income level and 52.5% lived in extended families; 46.7% of the patients said that they had the disease for 6-10 years. Eighty percent of the patients had glycosylated hemoglobin (HbA1c) >% 7; 60.0% were overweight and 82.5% had postprandial glucose (PBG) >180mg/dl.; 81.7% of the patients received insulin and diet treatment; 19.8% received diet and oral anti-diabetic (OAD) treatment; and 66.7% had DM complications. The mean TAS-20 score was 65.86±9.70; the mean DIF score was 24.20±5.61; the mean DDF score was 16.47± 3.19, and the mean EOT score was 25.18± 4.21 (Table-I).

**Table-I:** Mean alexithymia scores of elderly diabetic patients (n=120).

*Scale*	*X ± SD*	*Minimum*	*Maximum*
TAS-20	65.86 ± 9.70	38	89
DIF	24.20 ± 5.61	7	34
DDF	16.47 ± 3.19	5	23
EOT	25.18 ± 4.21	14	38

**Table-II:** Alexithymic characteristics of elderly diabetic patients (n=120).

*Alexithymic characteristics*	*Alexithymic*		*Nonalexithymic*		*Total*		p**
	*n*	*%*	*n*	*%*	*n*	%*	
*Sex*							
Female Male	62 29	68.1 31.9	17 12	58.6 41.4	79 41	65.8 34.2	0.347
*Age groups*							
60-69 70-79 80 ^+^	36 45 10	39.6 49.5 11.0	15 10 4	51.7 34.5 13.8	51 55 14	42.5 45.8 11.7	0.370
*Marital status*							
Single Married Widowed	12 67 12	13.2 73.6 13.2	1 23 5	3.4 79.3 17.2	13 90 17	10.8 75.0 14.2	0.321
*Educational status*							
Illiterate Literate Primary School High School	34 26 30 1	37.4 28.6 33.0 1.1	8 10 10 1	27.6 34.5 34.5 3.4	42 36 40 2	35.0 30.0 33.3 1.7	0.659
*Income status*							
Low Moderate High	29 54 8	31.9 59.3 8.8	2 23 4	6.9 79.3 13.8	31 77 12	25.8 64.2 10.0	0.027
*Professional status*							
Self employed Retired Housewives	2 39 50	2.2 42.9 54.9	1 15 13	3.4 51.7 44.8	3 54 63	2.5 45.0 52.5	0.622
*Family structure*							
Nuclear Family Extended Family	38 53	41.8 58.2	19 10	65.5 34.5	57 63	47.5 52.5	0.026
*Disease duration*							
0-1 year 2-5 years 6-10 years 11-15 years 16 years ^+^	2 14 44 22 9	2.2 15.4 48.4 24.2 9.9	2 11 12 3 1	6.9 37.9 41.4 10.3 3.4	4 25 56 25 10	3.3 20.8 46.7 20.8 8.3	0.038
*HbA1c*							
> %7 < % 7	73 18	80.2 19.8	23 6	79.3 20.7	96 24	80 20	0.915
*BMI*							
Normal Overweight Obese	4 55 32	4.4 60.4 35.2	3 17 9	10.3 58.6 31.0	7 72 41	5.8 60.0 34.2	0.483
*PBG*							
>180mg/dl <180mg/dl	75 16	82.4 17.6	24 5	82.8 17.2	99 21	82.5 17.5	0.966
*Treatment*							
Diet + OAD Insulin + diet	18 73	19.8 80.2	4 25	13.8 86.2	22 98	18.3 81.7	0.468
*DM complication*							
Yes No	62 29	68.1 31.9	18 11	62.1 37.9	80 40	66.7 33.3	0.546
*Total*	91	100.0	29	100.0	120	100.0	

*Column percentage, **Chi-square test value

Alexithymic characteristics of the study participants with DM were presented in Table-II. The female study participants in the 70-79 age group were married, illiterate, housewives, had HbA1c >7%, were overweight, had PBG >180mg/dl, had insulin and diet treatment and had DM complications and they presented higher alexithymic characteristics. However, there was not any statistically significant difference (p>0.05). We found a significant difference between alexithymic characteristics of the elderly with DM and their income levels, family types and disease duration.

## DISCUSSION

DM is a chronic disease that significantly impacts the lives of those affected and leads to physical, psychological and social adaptation problems.^10^

The study investigated alexithymic characteristics of elderly patients with DM. According to the TAS-20 mean scores, 75.8% of our diabetic elderly were found to be alexithymic. The TAS-20 subscales showed that our patients experienced above-average DIF and DDF, and they also had EOT styles more than the average population. Topsever et al. reported that 65% of their patients were alexithymic.^9^ Abramson et al. found patients with diabetes to be more alexithymic than the control group.^15^ Our study findings supported the results of that research.

Although the alexithymic characteristics of females were found to be higher than males, there was no significance. One study has reported that the alexithymic scores of females were higher than males.^9^ Similar to our study, two other studies of the elderly revealed that gender was an important factor in alexithymic characteristics.^16^^,^^17^

Other studies have also found that the elderly had more alexithymic characteristics.^7^^,^^18^ Similarly, although our study explored more alexithymic features among the 70-79 age group, this finding was statistically not significant. However, Mattila et al. pointed out that alexithymic characteristics increased with age. These were found in 4.7% of young people but had increased to 29.3% among the elderly.^7^

We could not find any significance between marital status and alexithymic characteristics. Similarly, Joukamaa et al. could not determine any significant correlation between marital status and alexithymic characteristics.^16^

Our finding that alexithymic characteristics were higher among the illiterate DM patients concurred with the fact that educational level is negatively correlated with alexithymic characteristics. These characteristics were 3.3% among those who had a higher level of educational status, whereas the rate was 16.5% among those with less education.^7^^,^^19^

There was a significant difference between income status of the patients and their alexithymic characteristics. Kokkonen et al. also found a correlation between low socio-economic status and alexithymia.^20^ Another study reported that TAS-20 scores of those with low- income levels were higher.^7^ Our study results also supported the correlation between income status and alexithymia. On the other hand, our investigation into the professional status of the patients did not support the opinion that profession played a role in the development of alexithymic characteristics.

We also explored the family structure of study participants. A significant difference was revealed between elderly patients with DM and alexithymia and living in a nuclear family and patients with higher alexithymic characteristics who were living in an extended family. Previous studies have also reported that alexithymic characteristics of those who lived with their spouses and other family members were higher. Although alexithymic individuals seem to enjoy interactions with others, the literature on this topic indicates that they make efforts to conceal their true feelings from others.^21^^,^^19^

A significant difference between alexithymic characteristics of the DM patients and disease duration was found. Disease duration affects daily living and emotions, and patients need time to learn how to adapt to and cope with the disease.^10^ We are of the opinion that once patients have had many years of learning to cope with DM, they become more knowledegable and sensitive in being able to identify, differentiate and express their physical and psychological symptoms.

No statistically significant correlation was found for those with alexithymic characteristics, HbA1c >7%, overweight issues, and a higher reading of PBG >180mg/dl. Sanden-Eriksson emphasized that there was a positive correlation between the HbA1c level and the patients who coped well with and adapted to the disease.^22^ Abramson et al. found that alexithymic characteristics were related to poor metabolic control and indicated that alexithymic characteristics negatively affected the diabetic patients’ ability to manage the disease. Furthermore, their inability to express their feelings made it difficult to regulate the glucose levels.^15^ Luminet et al. suggested that higher scores of DDF are correlated with poor glycemic control.^23^

Although alexithymic characteristics of the DM patients who received insulin and diet treatment were found higher in our study, there was no significant difference between the treatment and alexithymic characteristics. Topsever et al. also were unable to find a significant correlation between insulin treatment and alexithymia.^9^

DM complications occurred among 66.7% of our patients and the rate of alexithymic characteristics of the patients in whom DM complications occurred was higher in our study. In another study, the rate of DM complication occurrence was 43.0%, but DM complications and alexithymia were not found to be correlated.^9^

***Study Limitations:*** This study was conducted in only one city in Turkey. The results of this study may be generalized to the sample group in this study. The sample in this study reflects only one area of Turkey. The findings therefore cannot be generalized to all elderly patients with DM in Turkey. Thus, further studies with larger Turkish sample sizes are needed. However we believe that because our study is the first to investigate the alexithymic features of elderly patients with DM in Turkey, it will provide a foundation for future studies.

## CONCLUSION

Results of our study determined that the majority of the elderly with DM were found to be alexithymic. Income status, family structure and disease duration affected alexithymic characteristics of the DM patients. It is therefore clear that patients need information about the importance of being able to describe and express their emotions as this will help ensure that they receive the proper treatment in a timely manner.

## Notes

***Source of funding:*** Self financed study.

## References

[1] Bektas HA, Sahin H (2010). Daily life activity status and depression levels in geriatric patients. The J Academic Geriatrics.

[2] Colak R, Yabanoglu I, Torun E, Bayram F, Basturk M, Sofuoglu S (2003). Evaluation of psychiatric patients with diabetes mellitus. Diabetes Sci.

[3] Gorpe U (2008). Psychological problems in diabetes mellitus. I.U. Cerrahpasa Medical Faculty Continuing Medical Education Activities. Symposium Series of Common Psychiatric Disorders in Turkey.

[4] Kooiman CG, Bolk JH, Brand R, Trijburg RW, Rooijmans HG (2000). Is alexithymia a risk factor forum explained physical symptoms in general medical out patients?. Psychosom Med.

[5] Phillips LH, MacLean RDJ, Allen R (2002). Age and the understanding of emotions: neuropsychological and socio cognitive perspectives. J Gerontology: Psychological Sciences.

[6] Bamonti PM, Heisel MJ, Topciu RA, Franus N, Talbot NL, Duberstein PR (2010). Association of alexithymia and depression symptom severity in adults 50 years of age and older. Am J Geriatr Psychiatry.

[7] Mattila AK, Salminen JK, Nummi T, Joukamaa M (2006). Age is strongly associated with alexithymia in the general population. J Psychosom Res.

[8] Sapozhnikova IE, Tarlovskaia EI, Madianov IV, Vedenskaia TP (2012). The degree of alexithymia in type 2 diabetes mellitus patients and its association with medical and demographic parameters. Ter Arkh.

[9] Topsever P, Filiz TM, Salman S, Sengul A, Sarac E, Topalli R (2006). Alexithymia in diabetes mellitus. Scott Med J.

[10] Bahar A (2006). Determination of the level of anxiety and depression of diabetes mellitus patients. J Firat Health Services.

[11] Demirtas A, Akbayrak N (2009). The adaptation to their sickness in patients with type 2 diabetes mellitus. Anatol J Clin Investig.

[12] Kocak R (2002). Alexithymia: Theoretical framework therapeutic approaches and related researches. Ankara Uni J Faculty Educ Sci.

[13] Bagby RM, Parker JDA, Taylor GJ (1994). The Twenty-Item Toronto Alexithymia Scale-I; Item selection and cross-validation of the factor structure. J Psychosom Res.

[14] Sayar K, Gulec H, Ak I (2001). The reliability and validity of The Twenty-Item Toronto Alexithymia Scale.

[15] Abramson L, McClelland DC, Brown D, Kelner S, Jr (1991). Alexithymic characteristics and metabolic control in diabetic and healthy adults. J Nerv Ment Dis.

[16] Joukamaa M, Saarijarvi S, Muuriaisniemi ML, Salokangas RK (1996). Alexithymia in a normal elderly population. Compr Psychiatry.

[17] Gunzelmann T, Kupfer J, Brahler E (2002). Alexithymia in the elderly general population. Compr Psychiatry.

[18] Pasini A, Ceripa S, Ciani N (1992). Alexithymia as related to sex, age, and educational level: results of the Toronto Alexithymia Scale in 417 normal subjects. Compr Psychiatry.

[19] Yurt E (2006). Alexthymia in schizophrenia patients; negative sympoms, medication side effects, the relation between depression and insight. T.C. Ministry of Health Expertise Thesis.

[20] Kokkonen P, Karvonen JT, Veijola J, Laksyk K, Jarvelin MR, Joukamaa M (2001). Prevalence and sociodemographic correlates of alexithymia in a population sample of young adults. Compr Psychiatry.

[21] Kauhanen J, Kaplan GA, Julkunen J, Wilson TW, Salonen JT (1993). Social factors in alexithymia. Compr Psychiatry.

[22] Sanden-Eriksson B (2000). Coping with type-2 diabetes the role of sense of coherence compared with active management. J Adv Nurs.

[23] Luminet O, Timary P, Buysschaert M, Luts A (2006). The role of alexithymia factors in glucose control of persons with type 1 diabetes: a pilot study. Diabetes Metab.

